# Suture-based vs. pure plug-based vascular closure devices for VA-ECMO decannulation–A retrospective observational study

**DOI:** 10.3389/fcvm.2023.1106114

**Published:** 2023-01-26

**Authors:** Clemens Scherer, Hans Theiss, Mario Istrefi, Leonhard Binzenhöfer, Danny Kupka, Thomas Stocker, Enzo Lüsebrink, Era Stambollxhiu, Ahmed Alemic, Tobias Petzold, Konstantin Stark, Simon Deseive, Daniel Braun, Dominik Joskowiak, Sven Peterss, Jörg Hausleiter, Christian Hagl, Steffen Massberg, Martin Orban

**Affiliations:** ^1^Department of Medicine I, University Hospital, LMU Munich, Munich, Germany; ^2^German Center for Cardiovascular Research, Partner Site Munich Heart Alliance, Munich, Germany; ^3^Department of Medical Oncology and Hematology, University Hospital Zurich, Zurich, Switzerland; ^4^Department of Cardiac Surgery, University Hospital, LMU Munich, Munich, Germany

**Keywords:** cardiogenic shock, VA-ECMO, *ProGlide*, *MANTA*, suture-based closure device, plug-based closure device, veno-arterial extracorporeal membrane oxygenation

## Abstract

**Background:**

Veno-arterial extracorporeal membrane oxygenation (VA-ECMO) is a valuable treatment option for patients in cardiogenic shock, but complications during decannulation may worsen the overall outcome. Therefore, the aim of this study was to compare the efficacy and safety of suture-based to pure plug-based vascular closure devices for VA-ECMO decannulation.

**Methods:**

In this retrospective study, the procedural outcome of 33 patients with suture-based *Perclose ProGlide* closure devices was compared to 38 patients with MANTA plug-based closure devices.

**Results:**

Rate of technically correct placement of closure devices was 88% in the suture-based group and 97% in the plug-based group (*p* = 0.27). There was a significant reduction of severe bleeding events during VA-ECMO decannulation in plug-based versus suture-based systems (3% vs. 21%, *p* = 0.04). Ischemic complications occurred in 6% with suture-based and 5% with plug-based device (*p* = 1.00). Pseudoaneurysm formation was detected in 3% in both groups (*p* = 1.00). No switch to vascular surgery due to bleeding after decannulation was necessary in both groups.

**Conclusion:**

Based on our retrospective analysis, we propose that plug-based vascular closure should be the preferred option for VA-ECMO decannulation. This hypothesis should be further tested in a randomized trial.

## 1. Introduction

For patients with moderate to severe cardiogenic shock, veno-arterial extracorporeal membrane oxygenation (VA-ECMO) remains a valuable treatment option, although sufficient randomized controlled trials are missing to this day (1, 2). Complications of VA-ECMO therapy, such as severe bleeding during decannulation, may impact outcome of those patients. Previous studies analyzing complications of VA-ECMO reported vascular complications such as ischemia, bleeding, compartment syndrome and amputations in a relevant proportion (3).

*Perclose ProGlide* is a suture-mediated closure system and widely used for different interventions such as transcatheter aortic valve replacement and VA-ECMO decannulation (4, 5). *MANTA* is a novel plug-based closure device, which is specifically designed for large bore femoral arterial access site closure (6). In a recent study, the composite endpoint of vascular complications and wound infections for VA-ECMO decannulation was lower in the plug-based closure device group compared to patients undergoing surgical cannula removal (7).

To date, no larger study has ever compared the efficacy of suture-based vs. pure plug-based closure devices for VA-ECMO decannulation. Our study hypothesis was, that usage of plug-based closure devices may reduce bleeding complications during VA-ECMO decannulation.

## 2. Materials and methods

### 2.1. Study population

After approval by the local Ethics Committee (IRB number: 18-001) and registration at the WHO International Clinical Trials Registry Platform (DRKS00015860), patients with cardiogenic shock treated in the cardiac intensive care unit (ICU) of Ludwig-Maximilians-University (LMU) from 01/2010 until 11/2021 were included in the LMUshock registry in compliance with the Declaration of Helsinki and German data protection laws. Cardiogenic shock was defined by ESC guidelines (8), the IABP-SHOCK II trial (9) and CULPRIT SHOCK trial (10). Informed consent was obtained from patients involved in the study or their legal guardians.

### 2.2. Study endpoints

Primary study endpoints were closure device implantation success rate, defined as technically correct placement of closure devices, and rate of severe residual bleedings due to decannulation, defined as a bleeding event requiring an unplanned usage of a femoral compression device (FemoStop, St. Jude Medical, Libertyville Township, IL, USA) to stop active bleeding from the arterial cannula explantation site. Secondary study endpoints were rate of complications, use of femoral compression device after decannulation and rate of complications requiring a switch to open vascular surgery due to closure device failure.

### 2.3. VA-ECMO cannulation and decannulation

Veno-arterial extracorporeal membrane oxygenation implantation was guided by fluoroscopy or ultrasound to avoid a puncture distally from femoral artery bifurcation. However, heavy calcifications at the puncture site were a contraindication. Puncture sites for arterial and venous cannula were proximal femoral artery and vein. Antegrade perfusion sheath was inserted into the superficial femoral artery.

In our institution, decannulation is performed at bedside. From 02/2019 until 05/2020, *Perclose ProGlide* suture-based closure device (Abbott Cardiovascular, Plymouth, MA, USA) was exclusively used for VA-ECMO decannulation. Beginning from 06/2020 plug-based *MANTA* size 18F (Teleflex, Morrisville, NC, USA) was the primarily used closure device. However, two patients received a suture-based closure device in 08/2020 and 09/2020 due to the unavailability of the two plug-based closure device trained physicians.

Anticoagulation was stopped 4 h before planned decannulation. We use a standardized protocol for guide wire insertion into the arterial cannula as published previously (11). In the suture-based vascular closure group two devices were applied to achieve vessel closure. In case of focal calcification, which prevented the first or second attempt of suture-based closure device application, another attempt was undertaken with a different angle of insertion. In the pure plug-based vascular closure group vascular sonography was undertaken before decannulation to determine the distance from skin to vessel entry. After final removal of the venous and antegrade perfusion sheath manual compression was then continued for at least 5 min and puncture site covered by pressure bandage for 12 h in all patients.

### 2.4. Statistical analysis

Statistical analysis was performed with R (version 4.0.1, The R Foundation) following the Strengthening the Reporting of Observational Studies in Epidemiology (STROBE) statement (12). Normally distributed continuous variables were reported as mean with standard deviation and non-normally distributed continuous variables as median with interquartile ranges. *T*-test and Mann–Whitney-*U* test were used to compare groups, respectively. One-way analysis of variance and Kruskal–Wallis rank-sum test were used, respectively, to compare three or more groups. Categorical variables were reported as absolute numbers and percentages and Chi-squared or Fisher’s exact test was utilized for comparison. All tests were 2-tailed, and *p*-values < 0.05 were considered as significant.

## 3. Results

### 3.1. Study population and baseline characteristics

At time of analysis, 1,252 patients were included in the LMUshock registry. Of these, 368 patients with VA-ECMO treatment were available for analysis. Suture-based closure device was utilized in 33 patients as the first device, mainly between 02/2019 until 05/2020. Plug-based closure device was used in 38 patients beginning from 06/2020 (Supplementary Figure 1). There was no statistical difference between the suture-based and the plug-based device group with respect to baseline characteristics (Table 1).

**TABLE 1:** Baseline characteristics.

Variables	All patients (*n* = 71)	Suture-based Proglide Perclose (*n* = 33)	Plug-based MANTA (*n* = 38)	*P*-value
Age, years (SD)	58.2 (11.8)	58.6 (11.8)	57.9 (12.0)	0.81
Male gender, *n* (%)	55 (77.5)	24 (72.7)	31 (81.6)	0.54
Body mass index, kg/m^2^ (SD)	27.3 (3.8)	26.6 (3.4)	27.9 (4.0)	0.13
Previous PCI, *n* (%)	16 (22.5)	8 (24.2)	8 (21.1)	0.97
Previous CABG, *n* (%)	6 (8.5)	3 (9.1)	3 (7.9)	1.00
Previous stroke, *n* (%)	6 (8.5)	3 (9.1)	3 (7.9)	1.00
Known peripheral artery disease, *n* (%)	2 (2.8)	1 (3.0)	1 (2.6)	1.00
Cardiac arrest, *n* (%)	45 (63.4)	24 (72.7)	21 (55.3)	0.20
Out-of-hospital cardiac arrest, *n* (%)	18 (25.4)	10 (30.3)	8 (21.1)	0.54
Duration of cardio-pulmonary resuscitation if applicable, minutes [IQR]	15.0 [11.0, 33.0]	14.0 [10.8, 34.8]	15.0 [12.0, 30.0]	0.80
Cause of cardiogenic shock, *n* (%) Primary arrhythmia Decompensated CMP Myocarditis NSTEMI Other STEMI Valvular	4 (5.6) 17 (23.9) 5 (7.0) 13 (18.3) 3 (4.2) 28 (39.4) 1 (1.4)	0 (0.0) 5 (15.2) 3 (9.1) 10 (30.3) 1 (3.0) 14 (42.4) 0 (0.0)	4 (10.5) 12 (31.6) 2 (5.3) 3 (7.9) 2 (5.3) 14 (36.8) 1 (2.6)	0.06
Percutaneous coronary intervention, *n* (%)	39 (54.9)	21 (63.6)	18 (47.4)	0.26
ASA, *n* (%)	41 (57.7)	23 (69.7)	18 (47.4)	0.10
Clopidogrel, *n* (%)	4 (5.6)	2 (6.1)	2 (5.3)	1.00
Prasugrel, *n* (%)	27 (38.0)	15 (45.5)	12 (31.6)	0.34
Ticagrelor, *n* (%)	4 (5.6)	2 (6.1)	2 (5.3)	1.00
UFH, *n* (%)	69 (97.2)	32 (97.0)	37 (97.4)	1.00

Data are *n* (%), mean (SD), median [IQR].

PCI, percutaneous coronary intervention; CABG, coronary artery bypass graft; NSTEMI, Non-ST-elevation myocardial infarction; STEMI, ST-elevation myocardial infarction, CMP, cardiomyopathy; ASA, acetylsalicylic acid; UFH, unfractionated heparin.

### 3.2. ICU parameters

Almost all patients received therapeutic anticoagulation treatment with unfractionated heparin (97 vs. 97%, *p* = 1.00). Overall, antiplatelet therapy was not significantly different. However, numerically but not statistically more patients received acetylsalicylic acid and prasugrel in the suture-based group, which is in line with a higher proportion of patients with myocardial infarction. Duration of VA-ECMO treatment was comparable in both groups (3.9 vs. 4.7 days, *p* = 0.57). Mean arterial (16.4 vs. 16.7 Fr, *p* = 0.29) and venous sheath size (22.6 vs. 22.4 Fr, *p* = 0.43) was similar between both groups (Table 2). Amount of all used arterial sheath sizes are displayed in Table 3.

**TABLE 2:** VA-ECMO characteristics.

Variables	All patients (*n* = 71)	Suture-based Proglide Perclose (*n* = 33)	Plug-based MANTA (*n* = 38)	*P*-value
Duration VA-ECMO treatment in days, *n* (%)	4.1 [2.7, 7.1]	3.9 [2.7, 6.8]	4.7 [2.7, 7.4]	0.57
SAVE score (SD)	−7.0 (5.5)	−8.3 (4.9)	−5.9 (5.8)	0.07
eCPR, *n* (%)	11 (15.1)	4 (12.1)	7 (18.4)	0.69
Platelet count before decannulation in 10^9^/L, median [IQR]	77.0 [47.0, 107.0]	72.0 [43.5, 103.8]	77.0 [50.0, 108.0]	0.58
INR before decannulation, median [IQR]	1.1 [1.1, 1.3]	1.1 [1.1, 1.2]	1.1 [1.0, 1.3]	0.32
PTT before decannulation, median [IQR]	49.0 [37.0, 60.0]	48.5 [37.8, 58.2]	49.0 [35.0, 61.0]	0.78
Decannulation: device implantation success rate, *n* (%)	66 (93.0)	29 (87.9)	37 (97.4)	0.27
Decannulation: number of closure devices, median [IQR]		2.0 [2.0, 2.0]		
Decannulation: severe residual bleeding, *n* (%)	8 (11.3)	7 (21.2)	1 (2.6)	0.04
Decannulation: red blood cell transfusion, *n* (%)	31 (43.7)	17 (51.5)	14 (36.8)	0.32
Decannulation: ischemic complications, *n* (%)	4 (5.6)	2 (6.1)	2 (5.3)	1.00
Decannulation: pseudoaneurysm formation, *n* (%)	2 (2.8)	1 (3.0)	1 (2.6)	1.00
Decannulation: use of femoral compression system FemoStop, *n* (%)	13 (18.3)	9 (27.3)	4 (10.5)	0.13
Decannulation: switch to open surgery due to bleeding, *n* (%)	0 (0.0)	0 (0.0)	0 (0.0)	1.00
Decannulation: switch to open surgery due to ischemic complications, *n* (%)	3 (4.2)	2 (6.1)	1 (2.6)	0.90

Data are *n* (%), mean (SD), median [IQR].

VA-ECMO, veno-arterial extra corporeal membrane oxygenation; SAVE, Survival after Veno-Arterial ECMO; eCPR, extracorporeal cardiopulmonary resuscitation.

**TABLE 3:** VA-ECMO arterial sheath size.

Type of closure device	15 *F*	17 *F*	19 *F*
Suture-based Proglide Perclose (*n* = 33)	11	21	1
Plug-based MANTA (*n* = 38)	10	26	2

Absolute number of arterial sheath sizes used for patients with suture-based vs. plug-based closure devices.

### 3.3. Decannulation

Before decannulation, platelet count (72 vs. 77 × 10^9^/L, *p* = 0.58), International Normalized Ratio (INR, 1.1 vs. 1.1, *p* = 0.32) and partial thromboplastin time (PTT, 49 vs. 49 s, *p* = 0.78) were not statistically different between the suture-based and the plug-based group.

**FIGURE 1:**
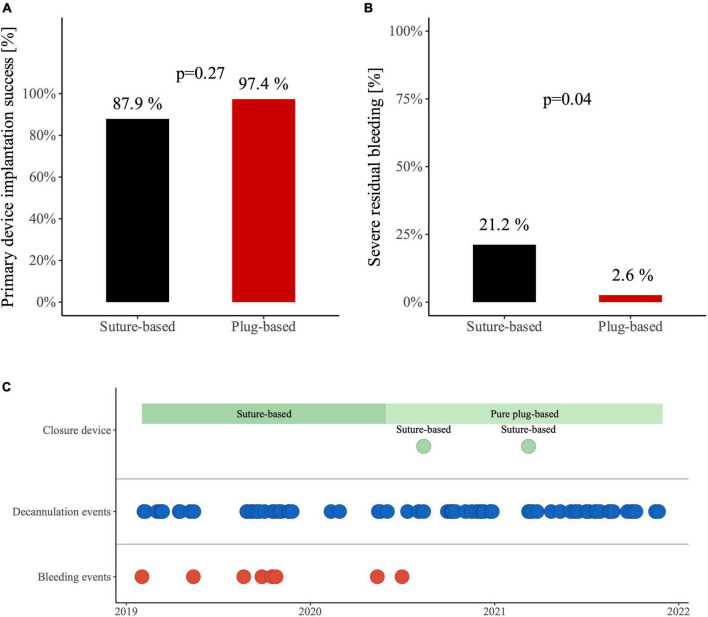
**(A)** Device implantation success for veno-arterial extracorporeal membrane oxygenation (VA-ECMO) decannulation with suture-based Proglide closure device and plug-based MANTA closure device. **(B)** Occurrence of severe residual bleeding after VA-ECMO decannulation in patients with suture-based Proglide closure device and plug-based MANTA closure device. **(C)** Distribution of severe bleeding events.

Closure device implantation success rate, defined as the technically correct placement of closure devices, was 88% in the suture-based group versus 97% in the plug-based group (Figure 1A, *p* = 0.27). Median number of devices used was two for patients with suture-based closure device and always one for patients with plug-based closure device (*p* < 0.01). Severe bleeding events were more frequent in the suture-based (21%) compared to the plug-based group (3%) (Figure 1B, *p* = 0.04) and were distributed evenly over the whole time period for the suture-based closure device group (Figure 1C).

Ischemic complications occurred in two patients with suture-based and two patients with plug-based device (6% vs. 5%, *p* = 1.00): one patient in the suture-based group developed an occlusion of the right femoral artery, probably due to heparin-induced thrombocytopenia type II, 4 days after decannulation, which was treated by embolectomy. For the second patient in the suture-based group, an asymptomatic occlusion of the right external iliac and femoral artery was detected coincidentally in Computed Tomography (CT) angiography 4 days after closure device use and the patient was treated by vascular surgery. In both patients, compression system FemoStop^®^ was used after decannulation due to bleeding. In the plug-based group, closure device was used in one patient with severe peripheral artery disease. Shortly after decannulation, arterial thrombosis was detected, which required vascular surgery. For the second patient in the plug-based group, a small arterial thrombosis was suspected 1 week after decannulation. No further action despite anticoagulation was initiated.

Pseudoaneurysm formation was detected in one patient in each group (3%, *p* = 1.00). In one case, this was successfully treated by thrombin injection. In the other case, suture of the vessel by vascular surgery was performed 1 week after decannulation.

Application of a femoral compression system was required in 27% of patient with suture-based closure device and 11% of patients with plug-based closure device (*p* = 0.13). No switch to vascular surgery due to bleeding after decannulation was necessary in both groups.

## 4. Discussion

This single-center, retrospective study compared the success and complications rates of VA-ECMO decannulation with suture-based *ProGlide* closure device and plug-based *MANTA* closure device. The main findings of our study are as follows: (1) Severe residual bleeding occurred more often in the suture-based group with 21% compared to the plug-based group with 3%, (2) rates of ischemic and vascular complications were comparable in both groups, (3) closure device implantation success rate was high in both groups, and (4) no switch to open vascular surgery due to bleeding after decannulation was necessary. The low rate of severe complications with both vascular closure devices demonstrates the overall safety of the percutaneous vessel closure approach.

We previously compared manual compression to suture-based closure for VA-ECMO decannulation and showed that femoral compression systems were more frequently used in the manual compression group (13). Moreover, vascular surgery due to bleeding events were performed numerically more often in the manual compression group compared to the suture-based group (11% vs. 0%) (13). Combining our previous work with the current data, a plug-based closure device should be the preferred option for save VA-ECMO decannulation to decrease rates of severe bleeding.

Our study contrasts the results of the CHOICE-CLOSURE randomized clinical trial, which compared suture-based vascular closure to plug-based vascular closure for patients treated with transfemoral transcatheter aortic valve replacement (14). In that trial, plug-based closure was associated with a higher rate of vascular complications. However, hemostasis time was very short in that trial with only 80 s in the plug-based group. In our cohort, manual pressure was applied for at least 5 min. Furthermore, rates of vascular complications were considerably lower in our study, which may be explained by the younger age and lower prevalence of peripheral vascular disease in our patient cohort compared to the CHOICE-CLOSURE trial.

A recent meta-analysis, including four observational and one randomized controlled trial, compared suture-based to plug-based device closure for large-bore access site management (15). In the meta-analysis, no difference was found concerning bleeding and vascular complications. However, incidence of closure device failure was higher in the suture-based group.

Even though baseline characters were balanced between the groups, this is a retrospective analysis with its inherent limitations. All patients were treated only at one large European ECMO center. As treatment periods differed between the two groups, changes in practice, not directly related to decannulation, may have influenced the results of our study. However, all decannulations were performed by closure device-experienced physicians and bleeding events were evenly distributed for the suture-based group. Furthermore, implantation success rate and complications of decannulation were documented at time of the procedure.

Based on our data and lacking evidence from other studies, we propose that plug-based vascular closure should be preferred for VA-ECMO decannulation. Our results cannot be generalized due to the single-center and retrospective design. This hypothesis should be further tested in a randomized trial.

## Data availability statement

Dataset available from the corresponding author on reasonable request.

## Ethics statement

The studies involving human participants were reviewed and approved by the Ethikkommission der Medizinischen Fakultät, LMU Munich, IRB-number: 18-001. The patients/participants or their legal guardians provided their written informed consent to participate in this study.

## Author contributions

CS and MO designed the study, interpreted the data, and wrote the manuscript. HT, MI, LB, DK, TS, EL, ES, AA, TP, KS, SD, DB, DJ, and SP collected the data and critically revised the manuscript. JH, SM, and CH interpreted data and critically revised the manuscript. All authors read and approved the final manuscript.
