# Supplementary figures and images for: Suture-based vs. pure plug-based vascular closure devices for VA-ECMO decannulation–A retrospective observational study
Source: Front Cardiovasc Med. 2023 Jan 26;10:1106114. doi: 10.3389/fcvm.2023.1106114 (PMC9908581; doi:10.3389/fcvm.2023.1106114)

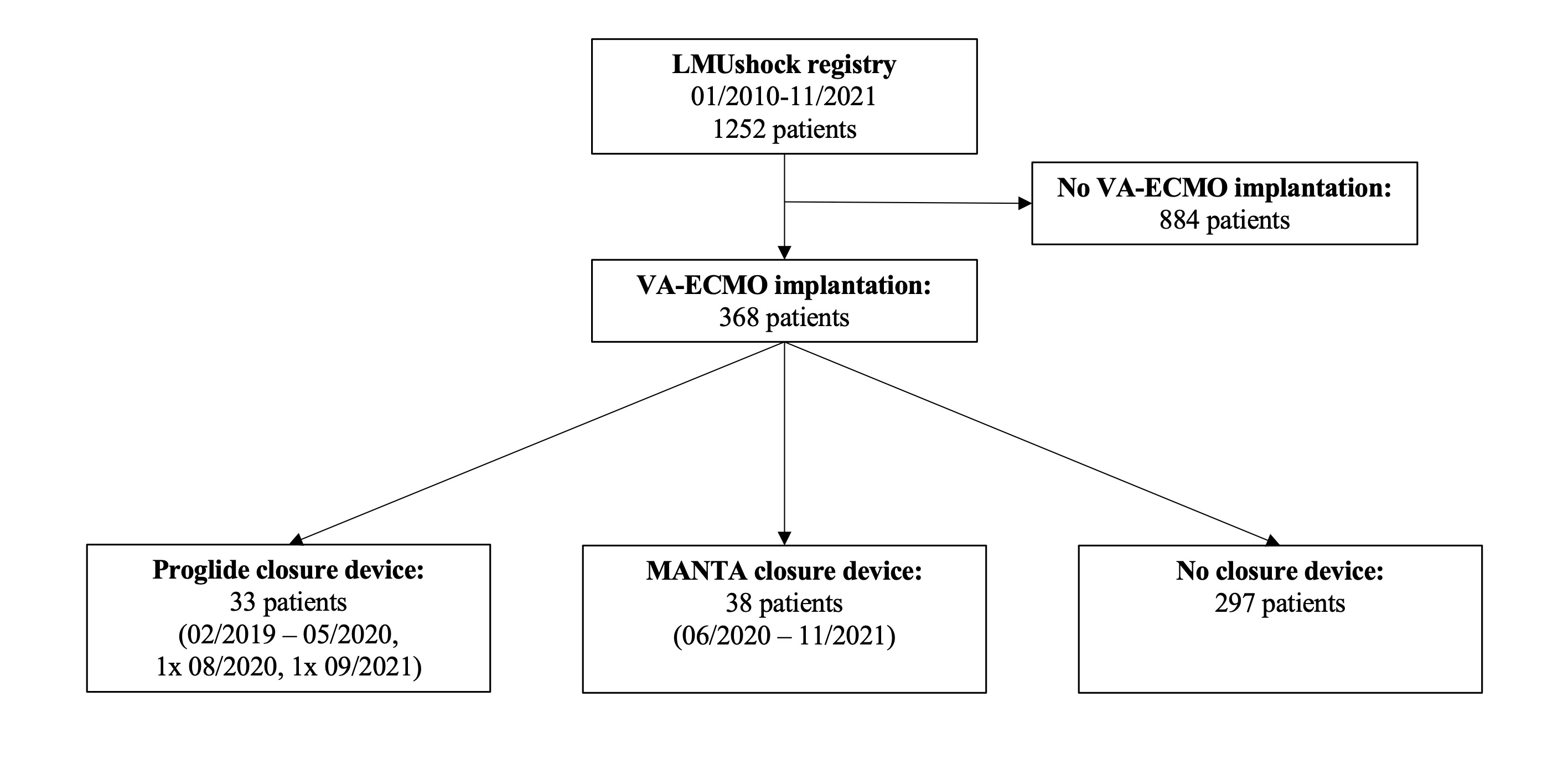

Supplement: Supplementary file 1 [file Image_1.JPEG]
